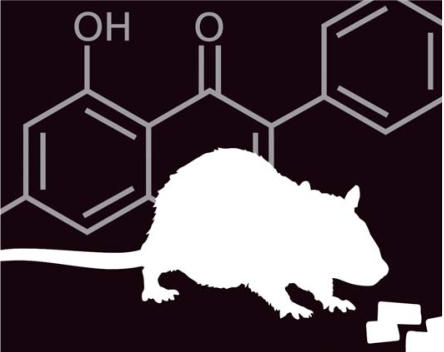# Dietary Dose: Rodent Feed Affects ED Screening Results

**Published:** 2007-12

**Authors:** Cynthia Washam

As the U.S. EPA begins its program to test endocrine-disrupting effects of pesticides, researchers caution that routine screening methods could distort results **[*EHP* 115:1717–1726; Thigpen et al.]**. The team discovered that some commercially available rodent diets can cause early sexual maturation similar to that induced by chemical endocrine disruptors. Furthermore, one rat strain commonly used by many researchers is not the most sensitive to the effects of endocrine disruptors and thus may not provide optimal results.

The U.S. EPA Endocrine Disruptor Screening Program was mandated by Congress in 1996 amid mounting evidence that hormone-mimicking chemicals in the environment alter sexual traits in exposed wildlife. Research suggests these chemicals might also contribute to increases in human male reproductive disorders including poor sperm quality, cryptorchidism, and hypospadias. In June 2007 the U.S. EPA published a list of 73 suspect chemicals for initial screening.

According to the authors of the current study, some rodent diets could distort screening results because they contain high levels of plant estrogens. The phytoestrogens genistein and daidzein are found in the soybeans used in many rodent diets. U.S. EPA guidelines allow a limited amount of genistein and daidzein in the diets of rodents used for screening. The authors report, however, that even approved levels of these compounds are sufficient to adversely impact sexual end points that researchers use to measure endocrine disruption. Furthermore, genistein and daidzein levels vary significantly among different batches of the same diet.

Rats on a diet with the highest genistein and daidzein concentrations reached sexual maturity several days earlier than those fed a different batch of the same diet containing lower levels of the compounds. In addition, rodents on high-calorie diets grew faster and reached sexual maturity earlier than those fed a low-calorie diet.

The researchers measured sexual maturity by observing the day each rodent’s vagina opened. Vaginal opening provides a non-invasive measurement that does not require the animal to be sacrificed. In contrast, sexual maturity is typically measured by uterine weight, which increases at puberty. The results revealed that dietary estrogens had a lesser effect on the vaginal opening day of Sprague-Dawley rats than on Fischer 344 rats or CD-1 mice. This indicates that the latter two species may be more sensitive to exogenous estrogens and thus are preferable for screening.

The authors call on scientists screening suspected endocrine disruptors to choose the most sensitive rodents, to minimize the animals’ exposure to dietary estrogens, and to control their caloric intake. Only then, they write, will scientists obtain results that are the most accurate, reproducible, and easiest to compare among laboratories.

## Figures and Tables

**Figure:**